# Elevated blood lipids are uncommon in patients with post-polio syndrome – a cross sectional study

**DOI:** 10.1186/s12883-015-0319-z

**Published:** 2015-04-29

**Authors:** Eva Melin, Thomas Kahan, Kristian Borg

**Affiliations:** Division of Rehabilitation Medicine, Department of Clinical Sciences, Danderyd Hospital, Karolinska Institutet, Stockholm, Sweden; Division of Cardiovascular Medicine, Department of Clinical Sciences, Danderyd Hospital, Karolinska Institutet, Stockholm, Sweden

**Keywords:** Post-polio syndrome, Neuromuscular disease, Dyslipidaemia, Cardiovascular risk

## Abstract

**Background:**

The post-polio syndrome occurs in people who previously have had poliomyelitis. After the initial recovery, new or increasing neurologic symptoms occur. Inflammation and dyslipidaemia may play an important role in the development of atherosclerotic complications, for example myocardial infarction and angina pectoris. Previous studies on cardiovascular risk factors in the post-polio syndrome have found a higher prevalence of hypertension, ischemic heart disease, hyperlipidaemia, and stroke in these patients. The present study was undertaken in order to evaluate whether post-polio patients have elevated lipid values, and if blood lipid abnormalities could be correlated to signs of inflammation.

**Methods:**

Cross-sectional study of 89 consecutive post-polio patients, (53 women, mean age 65 years) from the Post-Polio Outpatient Clinic, Danderyd University Hospital, Stockholm, Sweden. The lipid profiles of post-polio patients were compared to age and sex matched reference values from two earlier studies. Statistical analyses were performed by the Student’s *t*-test, and linear regression analyses were assessed by Pearson’s correlation coefficient.

**Results:**

Mean total cholesterol levels (5.7 mmol/L) were low or normal in post-polio patients, whereas low density lipoprotein levels (3.6 mmol/L) were normal, and high density lipoprotein (1.5 mmol/L) and triglycerides (1.4 mmol/L) lower than reference values. The prevalence of diabetes (7%), hypertension (38%), concomitant cardiovascular disease, (including angina pectoris, myocardial infarction, heart failure, atrial fibrillation and stroke) (7%), and calculated 10 year risk of coronary heart disease according to Framingham risk score algorithm (8%) was not increased in post-polio patients.

**Conclusions:**

Compared to reference populations, post-polio patients in Sweden appear to have low or normal total cholesterol and low density lipoprotein levels, whereas high density lipoprotein and triglyceride levels are low. Hence, a possible persisting inflammatory process in post-polio syndrome does not seem to be associated with increased lipids and an increased risk for coronary heart disease events.

## Background

The post-polio syndrome (PPS) is a condition occurring in people who previously have had poliomyelitis [1]. After an initial recovery, new or increasing symptoms occur, following a stable period of at least 15 years. Symptoms include muscle atrophy and weakness, muscle and joint pain, sensitivity to cold, fatigue, and dysphagia [1]. Due to their disability, PPS patients are less mobile than the normal population [2]. Decreased mobility could lead to weight gain and difficulties in physical exercise and maintenance of fitness. It has been assumed that this could have negative effects on the general health of PPS patients and increase the risk of cardiovascular disease, as seen in other disorders with muscle weakness, e.g. spinal cord injury [3]. Kang et al. [4] reported a higher prevalence of hypertension and ischemic heart disease in polio survivors in China. Dyslipidaemia was also described as comorbidity, which is in accordance with older studies in which dyslipidaemia was reported in as many as two thirds of a PPS population [5]. Thus, this could be a background and promote the development of atherosclerotic disease including ischemic stroke [5-7]. In contrast, however, a Norwegian study among people with previous poliomyelitis showed no increase in cardiovascular disease [8].

Increased levels of inflammatory markers in cerebrospinal fluid and in peripheral blood have been reported in PPS patients, and have led to the hypothesis of an ongoing inflammatory process [9,10]. Inflammation plays a key role in the atherosclerotic process, and elevated circulating markers of inflammation have been associated with an increased cardiovascular risk [11,12]. In chronic conditions with systemic inflammation, such as systemic lupus erythematosus and rheumatoid arthritis, altered metabolism of lipoproteins gives rise to changes in the lipid profile [13-15]. Even if PPS does not have the clinical hallmarks of a systemic inflammation, the hyperlipidaemia earlier described may be secondary to systemic inflammatory process. The proposed hyperlipidaemia has led to the suggestion that PPS patients should be treated generously with lipid lowering drugs. However, statins may give side effects as myalgia and muscular weakness, and less frequently rhabdomyolysis, which may be devastating for PPS patients regarding function and their ability to participate in designated rehabilitation programmes [16,17].

With this background, the present study was undertaken in order to evaluate whether dyslipidaemia is common in PPS patients and if so, if it is correlated to inflammatory markers. Furthermore, we attempted to estimate the risk of future coronary heart disease in PPS patients by means of the Framingham risk score [18].

## Methods

We recruited 89 consecutive patients (53 women and 36 men, mean age 65 ± 9 years) from the Post-Polio Outpatient Clinic at the Department of Rehabilitation Medicine, Danderyd University Hospital, Stockholm, Sweden. Patients were included on the basis of PPS diagnosis, based on the criteria given by March of Dimes, including neurophysiological examination. Exclusion criteria were patients who were unable to understand verbal or written instructions. Patient characteristics are presented in Table 1. Information on the patients was obtained both by questionnaire and from their medical files. In all, 17 patients were on lipid lowering medication (simvastatin 10-40 mg od or atorvastatin 10-40 mg od). All patients gave their informed consent according to the declaration of Helsinki, and the Regional Ethical Review Board, Stockholm, approved of the study.

**Table 1:** **Characteristics of the PPS patients included in the study**

	**Female**	**Male**	**All**
**n**	**53**	**36**	**89**
Age, years	66 (8)	64 (9)	65 (9)
Systolic blood pressure, mm Hg	139 (15)	139 (15)	139 (15)
HbA1c, %	4.7 (0.6)	4.8 (0.8)	4.7 (0.7)
Creatinine, μmol/L	56 (11)	66 (18)	60 (15)
C-reactive protein, mg/L	3.2 (2.3)	3.3 (2.2)	3.2 (2.2)
Erythrocyte sedimentation rate, mm	13.8 (8.8)	8.4 (5.5)	11.7 (8.1)
Leukocyte count, 10^9^/L	6.2 (1.6)	6.3 (1.6)	6.2 (1.6)
Hypertension %	40	33	38
Diabetes mellitus %	6	10	7
Cardiovascular disease %	8	6	7
Smoking %	8	3	5
Lipid lowering drug therapy %	15	25	19

Data are presented as mean values and standard deviations (SD) or proportions, as appropriate. Hypertension was defined as a blood pressure of 140/90 mm Hg or above, or on-going treatment with anti-hypertensive drugs. Patients who had an on-going pharmacological treatment for diabetes were cathegorised as having diabetes mellitus. Cardiovascular disease was defined as angina pectoris, myocardial infarction, heart failure, atrial fibrillation, and stroke.

Blood samples were collected in the morning after overnight fasting. Total cholesterol, high density lipoprotein cholesterol (HDL), and triglycerides, were analysed in serum, and low density lipoprotein cholesterol (LDL) was calculated by Friedewald’s formula [19]. The ratio between HDL and LDL (HDL/LDL) was calculated as it is commonly considered a marker of cardiovascular risk. Hyperlipidaemia was defined as total cholesterol of >4.9 mmol/L, or LDL >3.0 mmol/L, or on-going treatment with lipid lowering drugs. C-reactive protein, erythrocyte sedimentation rate, and leukocyte count were measured as markers of inflammation, in addition to creatinine and HbA1c. All the samples were analysed by standard techniques at accredited regional laboratories.

Lipid data were compared to reference values provided by two earlier published studies. The Nordic Reference Interval Project (NORIP) is based on blood samples from 3036 healthy individuals in the Nordic countries and provides reference data for total cholesterol, LDL, HDL, and triglycerides [20]. Jungner et al. reported on a Swedish population sample of 147576 males and females obtained from general health screening, with no information on the use of medication, and provides results for total cholesterol and triglycerides [21]. The results of our patients were matched for age and gender. In the study by Jungner et al., patients were divided into age categories of 10-year intervals, and divided into male and female, giving us the opportunity to match our patients to this. In the NORIP study the patients were divided into age categories of 18-29, 30-49 and ≥50, when the authors concluded that this was necessary (i.e. LDL and total cholesterol), and into male and female, allowing us to match our patients to this. When NORIP was used as reference, we separately evaluated data for all 89 PPS patients and for the 72 PPS patients who did not receive lipid lowering drugs. When the Jungner et al. study was used as reference, all 89 PPS patients were included, since that study did not exclude subjects on medication.

In 64 patients (41 women, mean age 64 ± 8 years, and 23 men, mean age 64 ± 10 years) with no known coronary heart disease and no therapy for high blood cholesterol the Framingham risk score was used to calculate the 10 year risk of developing an acute myocardial infarction and/or coronary death [18]. In 5 patients sufficient background data could not be obtained, and 3 patients were too old for the Framingham risk algorithm. This risk algorithm is based on age, gender, total cholesterol or LDL, HDL, blood pressure, diabetes mellitus, and smoking in adults more than 30 years old, with no known coronary heart disease.

### Statistics

Data are presented as mean values and standard deviations (SD). Statistical analyses were performed by the Student’s *t*-test, and linear regression analyses were assessed by Pearson’s correlation coefficient. A probability (p) <0.05 was considered statistically significant.

## Results

Age and smoking habits, as well as laboratory parameters and concomitant disorders are presented in Table 1. A minority of the patients were smoking. A history of cardiovascular disease (i.e. angina pectoris, myocardial infarction, heart failure, atrial fibrillation and stroke) was present in 7% of the patients. Hypertension was found in 38% of the patients, and the mean systolic blood pressure was normal but in the higher range. C-reactive protein and leukocyte count were normal, indicating that there was no on-going inflammation.

Serum lipid values are given in Table 2. Out of the 89 patients, 66 had hyperlipidemia following the definition, presented above (see Methods). However, compared to the reference populations, dyslipidaemia was less common in the PPS group. When analysing mean values, total cholesterol was lower in the PPS group than in the study by Jungner et al., but similar to the findings in NORIP. LDL levels were not different between the populations, whereas, HDL was lower in the PPS group than in NORIP. The PPS population had a LDL/HDL ratio of 2.6 (SD 0.9), as compared to a significantly lower ratio of 2.2 (SD 0.2) in NORIP (p = 0.03). In the PPS patients with no lipid lowering medication, the LDL/HDL ratio was 2.7 (SD 0.9). In the patients with medication for high blood lipids, the LDL/HDL ratio was 2.4 (SD 0.7). Triglyceride levels were lower in the PPS group than in the two reference populations.

**Table 2:** **Lipid values in the PPS patients and two reference populations**

	**Patients without lipid lowering drugs**			**All patients**					
	**PPS**	**NORIP**	**p**	**PPS**	**Jungner et al**	**p**	**PPS**	**NORIP**	**p**
Total cholesterol, mmol/L, all patients	5.9(1.0)	5.9	0.8	5.7(1.0)	6.4	<0.001	5.7(1.0)	5.9	0.09
Total cholesterol, female	6.1(0.9)	5.9	0.07	6.0(1.0)	6.5	0.001	6.0(1.0)	5.9	0.7
Total cholesterol, male	5.5(0.9)	5.9	0.04	5.3(0.9)	6.1	<0.001	5.3(0.9)	5.9	0.001
LDL, mmol/L, all patients	3.8(0.9)	3.6	0.10	-	-	-	3.6(0.9)	3.6	0.9
LDL, female	4.0(0.9)	3.6	0.03	-	-	-	3.8(1.0)	3.6	0.4
LDL, male	3.6(0.8)	3.6	0.8	-	-	-	3.4(0.8)	3.6	0.1
HDL, mmol/L, all patients	1.5(0.4)	1.7	0.001	-	-	-	1.5(0.4)	1.7	<0.001
HDL, female	1.6(0.4)	1.8	0.01	-	-	-	1.6(0.4)	1.8	<0.001
HDL, male	1.3(0.4)	1.5	0.02	-	-	-	1.3(0.4)	1.5	0.003
Triglycerides, mmol/L, all patients	1.3(0.6)	1.5	0.003	1.4(0.7)	1.6	0.05	1.4(0.7)	1.5	0.05
Triglycerides, female	1.2(0.6)	1.5	0.03	1.4(0.8)	1.5	0.5	1.4(0.8)	1.5	0.2
Triglycerides, male	1.4(0.7)	1.5	0.3	1.4(0.6)	1.7	0.007	1.4(0.6)	1.5	0.1

Total cholesterol, low density lipoprotein cholesterol (LDL), high density lipoprotein cholesterol (HDL), and triglyceride levels in all 89 PPS patients and in 72 PPS patients without lipid lowering medication, as compared to age and gender matched reference mean values, from NORIP and the study by Jungner et al. Mean value and standard deviation (SD).

There were no relations (all r^2^ values <0.03) between inflammatory markers (C-reactive protein, or sedimentation rate, or leukocyte count) and blood lipids (total cholesterol, LDL, HDL, or triglycerides).

The Framingham risk score calculated for the PPS patients, gave a 10-year mean risk of 8% for a coronary artery disease event.

## Discussion

The present study shows that patients with PPS have normal or lower total cholesterol values than healthy reference populations, matched for age and sex [20,21]. LDL values in the PPS patients were similar to those of the reference population. Hence, our results do not corroborate earlier findings of elevated total cholesterol levels in PPS patients [5,6]. HDL values were, however lower in patients with PPS, and the LDL/HDL ratio accordingly increased. In contrast to earlier findings [5,6] our results do not suggest that abnormal blood lipid values are common in PPS patients.

High triglyceride values are a common finding in patients with impaired glucose control. However, we observed lower triglyceride levels in the PPS group than in the reference populations. Furthermore, 7% of the PPS group had diabetes mellitus. This is comparable to subjects of similar age and sex in the same geographic region, as 7% of women and 10% of men aged 65 years in Stockholm were recently reported to have diabetes [22]. This suggests that patients with PPS do not have an increased prevalence of glucose homeostatic abnormalities.

Only few (7%) of the PPS patients had a history of cardiovascular disease (which included angina pectoris, myocardial infarction, heart failure, atrial fibrillation and stroke). For comparison, in patients of similar age (65 years) the prevalence of coronary heart disease in Denmark was reported at 9% [23], heart failure in Sweden at 2% [24], diagnosed atrial fibrillation in Sweden 4% [25], and stroke in Norway at 1% [26]. The risk of dying from heart disease each year in Sweden is about 200 per 100 000 inhabitants in women and 350 per 100 000 inhabitants in men [27]. Furthermore, the calculated Framingham risk score in the PPS group suggests an 8% 10 year risk for future coronary artery disease event. A 9-11% risk is considered low risk and 21-25% corresponds to normal risk according to the Framingham risk score [18]. Notwithstanding that we observed lower HDL in the PPS group, and low HDL is associated with an increased risk for cardiovascular events, HDL is actually included in the Framingham risk score. Taken together our findings are in agreement with results based on PPS patients and their siblings, presented by Farbu and collaborators [8], which suggest that PPS patients have a low prevalence of cardiovascular events. Regular contacts with Post-polio out-patient clinics could be beneficial for early diagnoses and may lead to reduction of risk factors.

We found no relation between markers of inflammation and lipid levels in patients with PPS. Our findings do not suggest that the inflammatory process in PPS will cause elevated lipid levels. In other chronic disorders with systemic inflammation, hyperlipidaemia is present [13-15]. In PPS signs of inflammation have been reported in peripheral blood, and in blood vessels of muscle tissue, which may be an indication of a systemic inflammation. However, PPS does not show other signs of cronic inflammation, as seen in for example systemic lupus erythematosus and rheumatoid arthritis. With the present results one may conclude that the inflammation in PPS has another background than the one producing hyperlipidaemia in chronic rheumatological diseases.

Our results provide no support for a generous approach in offering PPS patients treatment with statins. We suggest that statin treatment should be offered to PPS patients based on the same judgement as for other subjects. This may be particularly relevant in the rehabilitation of PPS patients, as statins have common side effects from skeletal muscle.

Some potential limitations of this study should be considered. First, low or normal cholesterol values and low triglyceride values may at first seem surprising, considering the physical disabilities and, thus, an assumed low physical activity of PPS patients. However, PPS patients use more energy for moving around than a normal population, making PPS patients actually more fit than might be expected [28]. This could, at least in part, contribute to our findings. Second, one may argue that the study population is not representative for PPS patients in general. However, our study population of consecutive patients is large, and PPS patients of all ages and with different degrees of disability attend our institution. When evaluating the patients also regarding age, gender, ethnicity and health status, we believe that the patients included in the study are representative. Third, the Framingham risk score could only be calculated for the PPS patients without concomitant coronary heart disease, which might give the impression of a lower risk for the PPS population, than they actually have. However, most PPS patients were free of cardiovascular disease and the prevalence of cardiovascular disorders appeared lower than in the general population suggesting that the possible confounding influence was limited.

Fourth, in previous studies cytokines were assessed as inflammatory markers. In the present study in clinical setting cytokine measurements were not available. Since blood lipid values in the PPS group were normal, we do not think that cytokine measurement would change the conclusion of the present study.

Finally in this study, we were not able to match the patients for exactly the same age as the controls. However, the lipid values in the reference material were presented in different age groups, giving us the opportunity to have a fair match of patients and controls.

## Conclusion

We found low or normal levels of total cholesterol, LDL, and HDL among PPS patients in Sweden. The inflammatory process in PPS does not seem to be associated with increased lipids values, and PPS does not confer an increased risk for cardiovascular disease. Thus, statin treatment should be offered to PPS patients on the same indication as in other subjects.
